# Nose-to-Brain Delivery of Chitosan-Grafted Leciplexes for Promoting the Bioavailability and Antidepressant Efficacy of Mirtazapine: In Vitro Assessment and Animal Studies

**DOI:** 10.3390/ph18010046

**Published:** 2025-01-03

**Authors:** Amani M. El Sisi, Essam M. Eissa, Ahmed H. E. Hassan, Marina A. Bekhet, Fatma I. Abo El-Ela, Eun Joo Roh, Rasha M. Kharshoum, Adel A. Ali

**Affiliations:** 1 Department of Pharmaceutics and Industrial Pharmacy, Faculty of Pharmacy, Beni-Suef University, Beni-Suef 62514, Egypt; amani_mcc@yahoo.com (A.M.E.S.); essam.mohamed@pharm.bsu.edu.eg (E.M.E.); rasha0mohd@hotmail.com (R.M.K.); adel.ali@pharm.bsu.edu.eg (A.A.A.); 2 Department of Medicinal Chemistry, Faculty of Pharmacy, Mansoura University, Mansoura 35516, Egypt; 3 Department of Pharmacology, Faculty of Veterinary Medicine, Beni-Suef University, Beni-Suef 62511, Egypt; fa.pharma@yahoo.com; 4 Chemical and Biological Integrative Research Center, Korea Institute of Science and Technology (KIST), Seoul 02792, Republic of Korea; 5 Division of Bio-Medical Science & Technology, University of Science and Technology, Daejeon 34113, Republic of Korea

**Keywords:** mirtazapine, depression, cationic leciplexes, nose-to-brain targeting, in vivo study, pharmacodynamics

## Abstract

**Background/Objectives**: Mirtazapine (MRZ) is a psychotropic drug prescribed to manage serious sorts of depression. By virtue of its extensive initial-pass metabolic process with poor water solubility, the ultimate bioavailability when taken orally is a mere 50%, necessitating repeated administration. The current inquiry intended to fabricate nose-to-brain chitosan-grafted cationic leciplexes of MRZ (CS-MRZ-LPX) to improve its pharmacokinetic weaknesses and boost the pharmacodynamics aspects. **Methods**: Primarily, MRZ-loaded leciplexes (MRZ-LPXs) were fabricated and tailored employing a central composite design (CCD). Vesicle diameter size (VS), entrapment efficiency (EE %), cumulative MRZ release percentage (CMRZR %), and total quantity penetrating after twenty-four hours (Q24) were the four parameters assessed. Then, the determined optimum formulation was coated with chitosan (CS-MRZ-LPX) and utilized in pharmacodynamics investigations and in vivo biologic distribution studies in Wistar male rats. **Results**: The customized MRZ-LPX formulation had a diameter size of 186.2 ± 3.5 nm and drug EE of 45.86 ± 0.76%. Also, the tailored MRZ-LPX formulation had a cumulative amount of MRZ released of 76.66 ± 3.06% and the total Q24 permeated was 383.23 ± 13.08 µg/cm^2^. Intranasal delivery of the tailored CS-MRZ-LPX revealed notably superior pharmacokinetic attributes inside the brain and circulation compared to the orally administered MRZ suspension and the intranasal free drug suspension (*p* < 0.05); the relative bioavailability was 370.9% and 385.6% for plasma and brain, respectively. Pharmacodynamics’ and immunohistopathological evaluations proved that optimum intranasal CS-MRZ-LPX boosted antidepressant activity compared to the oral and free nasal drug administration. **Conclusions**: CS-MRZ-LPX tailored formulation can potentially be regarded as a prospective nano platform to boost bioavailability and enhance pharmacodynamics efficacy. Ultimately, intranasal CS-MRZ-LPX can be considered a promising avenue for MRZ targeted brain delivery as an antidepressant.

## 1. Introduction

Depressive disorders are a severe mental plight striking approximately 4 percent of population and are an established root of debilitation around the world. These disorders are frequently linked to genetics, sociology, physical disorders, and biological reasons [1]. Depression is triggered by an aberration in monoaminergic neurotransmitter receptor occupancy (serotonin, norepinephrine, and dopamine) [2].

Mirtazapine (MRZ), deemed an antidepressant, is prescribed to manage moderate to severe dolefulness, and is authorized as a stand-alone tetracyclic antidepressant by the FDA. Referring to its mechanism of action, MRZ alleviates anxiety by boosting central noradrenergic and serotonergic (5-HT1) neurotransmission [3]. Despite the notion that MRZ is rapidly absorbed after oral consumption, its absolute bioavailability is only 50% due to its first-pass metabolism [4,5]. Furthermore, the therapeutic effectiveness of centrally acting medicines such as MRZ hinges on their extended presence at the site of action (the brain). Beyond that, simply improving oral bioavailability is inadequate [6]. An alternative MRZ targeted delivery strategy is a prerequisite for tackling the aforementioned challenges and enhancing brain bioavailability. 

Remarkably, intranasal delivery has sparked attention as a possible alternative way of attaining boosted medication absorption along with an enticing systemic therapeutic effect [7]. Intranasal delivery provides an ample absorptive region, a highly vascularized endothelial surface, facile application, and an initial-pass metabolism revocation, as well as the aptitude to guarantee swift and forthright penetration through a nasal passage [8]. A range of techniques are being investigated for augmenting intranasal medication delivery involving the assembly of vesicular platforms. Nano paradigms represent urgent advances in medication delivery involving tiny and bulk moieties through the nasal route by overcoming nasal delivery hurdles such as mucociliary elimination [9]. It has formerly been elucidated that chitosan-grafted PLGA nanoparticles promote duloxetine Hcl penetration via the nose, and this tailored formulation enhanced the drug’s brain bioavailability [10]. Furthermore, in citicoline research, intranasal niosomes imparted higher brain drug uptake than free intranasal and oral drug solutions [11]. Alsaidan, O.A. et al., in their research on venalafaxine transbiliosmes thermo gel using bio distribution analysis, showed that their formulation delivered intranasally had a relative bioavailability of 441% in the brain and 288% in plasma [12]. In this study, leciplexes (LPXs) were tailored and appraised as a novel platform for MRZ intranasal brain delivery.

LPX is a self-assembled nanocarrier commonly used to proficiently apply hydrophobic medicines [13]. The distinct advantage of leciplex over typical vesicular systems is the ease of preparation since it is a one-step fabrication procedure that generates nanosized vesicular assemblies by simple mixing; it also has the benefits of scalability, lack of organic solvent, and inherent improved stability [14]. LPX is a positively charged phospholipid-based vesicular system with a cationic surfactant and a biocompatible solvent as primary components. The positive charge of LPX enhances nanocarrier attachment onto negatively charged mucosal cell surfaces and increases cellular absorption of the loaded medication, which is why it was chosen to boost the nasal permeability of MRZ [15]. To increase drug penetration through nasal mucosa and prolong the residence time of the LPX formulation, chitosan (CS) was used to coat the MRZ-LPX tailored formulation as a mucoadhesive polymer. LPXs improved different drugs’ permeability and bioavailability in earlier studies [16,17]. In the literature, Trifluoperaizne-loaded LPX was studied as a potential nasal delivery system to treat depression [18]. Also, several previous studies examined different intranasal cationic nano formulations [19,20].

There are no earlier studies of the efficiency of LPX for the encapsulation and brain targeting of MRZ through the intranasal route. The novelty of the present inquiry is the assessment of the potential adaptation of this unique system, LPX, to achieve efficient nose-to-brain MRZ delivery with the aim of boosting bioavailability and sustaining antidepressant leverage, while minimizing the accompanying unwanted constraints for the orally administered dosage. From that specific perspective, LPX preparations were customized and subjected to in vitro characterization. The optimum formula was then fabricated with a chitosan coating (CS-MRZ-LPX). Nasal tolerability and toxicological studies on animals were performed to exclude any potential detrimental impacts. Ultimately, the ideal intranasal CS-MRZ-LPX formulation’s in vivo pharmacokinetic and pharmacodynamic behavior in rats was assessed in comparison to the corresponding intranasal free and oral MRZ suspensions.

## 2. Results and Discussion

### 2.1. Experimental Design and Optimization

**Figure 1:**
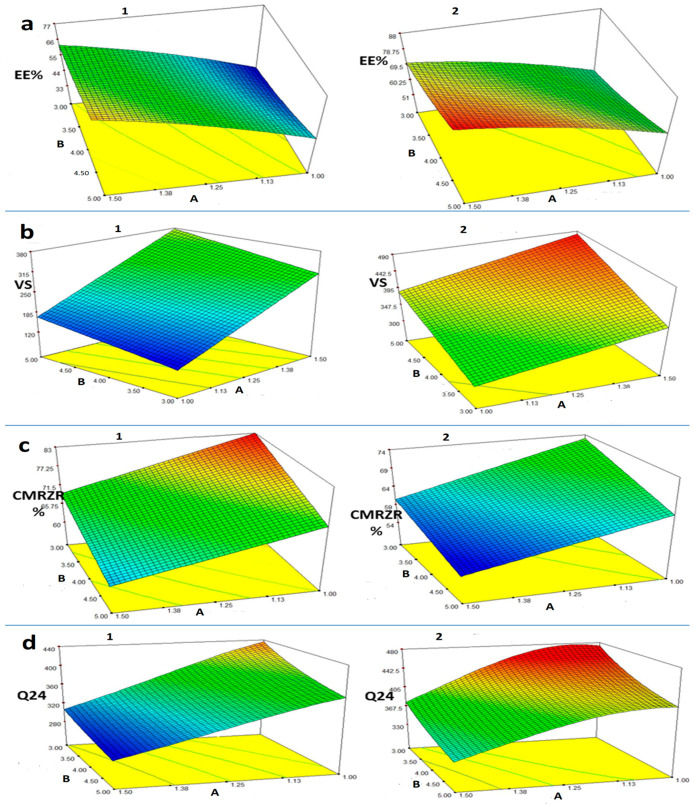
The 3D surface response diagrams showing the contribution of the three variables on (**a**) EE%, (**b**) VS, (**c**) CMRZR%, and (**d**) Q24, where (1) CTAB, (2) DDAB, (A) PL90G CONC, and (B) PL90G:SAA ratio.

CCD is a response surface methodology approach that significantly reduces the number of overall trials necessary in studies featuring categorical variables. Moreover, it facilitates the identification of the main and interactive effects of the component factors in a mixture while minimizing the predicted run discrepancies [21,22]. Following evaluation, the anticipated R^2^ values were in close proximity to the amended R^2^, suggesting that the model is appropriate. The contribution of PL90G (phospholipon 90G) concentration, PL90G: SAA ratio, and SAA (surfactant) type on LPX-EE %, VS, CMRZR%, and Q24 is readily apparent in a three-dimensional response surface graph (Figure 1). The design variables with their minimum and maximum levels, as well as the responses implemented in the study, are represented in Table 1. Supplementary Table S1 displays the coefficient of determination (R^2^), adjusted (R^2^), projected (R^2^), and CV% data.

### 2.2. Characterization of MRZ-LPX Formulations

#### 2.2.1. MRZ Entrapment Efficiency

The MRZ-EE% data demonstrated considerable disparity, comprising 33.1 ± 2.55% at the minimum to the maximum of 86.43 ± 3.15% (see Table 2). The EE responses were deemed most appropriately conveyed through the quadratic model (*p* < 0.0001), which was evidenced by a high F-value (88.54) with no insufficiency of fit (*p* = 0.0645; *p*-value > 0.05). The quadratic impact is frequently considered the most effective model for attaining maximum impact due to its integration of all independent factors, in isolation and in combination. The following is the expression of the second-order polynomial equation that shows the association between the uncorrelated factors on EE% (Y1):

(1) $$EE\%=+62.72+12.67A+7.45B+7.12C-2.37AC\;-2.56A^{2}\;$$

A positive sign indicates the synergistic impact of the factor (A, B, or C) on EE%, while an antagonistic effect is seen when the factor has negative sign. Y1 was significantly impacted (*p* < 0.0001) by the concentration of PL90G (A), having a positive sign, whereas EE% of F3 was 44.4 ± 3.12% and increased to 77.33 ± 1.76% in F11 when A spiked from 1% to 1.5%; however, both formulations utilized CTAB as a surfactant in constant proportions. A pair of theories might explain these findings. Essentially, the surface structure of PL90G may promote the creation of strong, consistent layers encasing MRZ, thereby decreasing leakage, as concurred by Date et al. [13]. Moreover, implementing a larger PL90G concentration would result in a higher viscosity, potentially hindering MRZ external diffusion and elevating its level [23].

Similarly, the positive effect of the molar ratio of PL90G: cationic SAA (B) exerted a significant synergistic effect on EE% (*p* < 0.0001). This correlation may result from SAA solubilizing lipids, triggering drug leakage from LPXs when their molar ratios are the same. Nevertheless, as the concentration of PL90G escalates, SAA’s ability to dissolve PL90G diminishes, leading to densely packed bilayers and, consequently, an upsurge in EE% [24]. The conclusions presented here align with the study conducted by Salama et al., which exhibited a notable decline in spironolactone encapsulation as the level of surfactant increased [25]. By altering the type of SAA (C), EE% was enhanced. This is due to the expanded lipophilicity of DDAB, which enabled the water-insoluble substance to be captured more efficiently [13,26]. Variations in EE% capacities of CTAB and DDAB are caused not only by their lipophilicity, but also by their chemical builds. DDAB has a double-alkyl-chain structure, which increases the accessible surface area to capture more MRZ molecules relative to CTAB’s single-alkyl-chain structure [27,28]. The 3D figure in Figure 1a shows how the three factors (A, B, and C) affect Y1.

#### 2.2.2. VS Analysis

A crucial physicochemical aspect of nanodrug delivery strategies is vesicle size, which impacts the nanosystem’s biological distribution, circulation half-life, and cellular utilization. In response to this fact, tiny VS may undergo more comprehensive absorption compared to larger ones [29]. By manipulating the factor levels within their respective constraints, the VS values of MRZ-LPXs spanned from 124.2 nm to 483.33 nm (Table 2), and the formulations also acquired a satisfactory polydispersity index (PDIs). PDI ranging <0.8 in Supplementary Table S2 signified commendable homogeneity. The optimal model identified to align with the VS data without any notable lack of fit value, *p* = 0.0745, was the 2FI model, exhibiting 201.85 (*p* < 0.0001) as the F-value. The polynomial equation shown below demonstrates an accurate correlation between Y2 and distinct variables:

(2) VS=+317.01+70.94A+37.7B+71.91C−27.73AC+8.66BC

The ANOVA results and the model equation demonstrate remarkable collaboration impacts of all factors on VS of MRZ-LPXs (*p* < 0.0001). All the three factors had a positive synergistic effect on VS. The upsurge in PL90G amounts causes larger vesicles to arise because they become stiffer. This finding is linked to EE% results, in which a substantial increase in lipid concentration corresponds to higher drug concentrations and a proportional expansion in diameter. Adding a cationic surfactant to LPX formulations notably dropped VS due to the steric repulsion manifested by the surfactant molecules; thus, vesicle consolidation was impeded or diminished. Another theory might pertain to a reduction in the aqueous–lipid interfacial tension, which causes smaller vesicles to form, or surfactant-mediated lipid solubilization, which causes drug leakage from LPXs and minimal VS [26]. These findings corroborate those of Khatoona et al. [30]. When factoring in C, vesicles made by the single-chained cationic CTAB were smaller than those made by the double-chained DDAB. The 3D figure in Figure 1b shows how the three independent variables (A, B, and C) affect Y2.

#### 2.2.3. In Vitro Release Evaluation

Based on the ANOVA test, the 2F interaction model adequately coincides with the CMRZR% data. A close association between the experimental and anticipated findings is also evidenced by the high R^2^ value of 0.9913 (Supplementary Table S1). Supplementary Figure S1 displays the in vitro profiles of MRZ release for both the unbound drug suspension and the MRZ-LPX formulations. The different LPX formulations inevitably expanded the release of MRZ (from 55.91 ± 2.48 to 82.93 ± 2.87% over 12 h). This verdict aligned with preceding MRZ inquiries [31,32]. Yet, when compared to previous research, the results show that the MRZ-LPX preparations used in this study exhibited better-sustained release patterns and higher levels of CMRZR% [4,33]. The polynomial equation below depicts the association between % CMRZR and the independent variables:

(3) % CMRZR=+67.22−5.93A−4.22B−3.95C+0.63AB+0.66BC 

As apparent by the negative signs in the equation, the concentration of PL90G (A), the molar ratio of PL90G: SAA (B), and the type of SAA (C) all had an unfavorable detrimental impact on drug release. The diffusion of drugs from vesicles decreased as the concentration of lipids rose, presumably due to a surge in stiffness [18,34]. Concerning (B), adopting a low PL90G: SAA molar ratio resulted in an abundance of diminutive vesicles. This resulted in an increase in surface area and a higher proportion of CMRZR [35]. When evaluating F9, F10, and F11, all of which contain the same concentrations of A and CTAB as SAA, it is clear that the release values drop as the ratio rises. The more lipophilic surfactant DDAB resulted in a reduction in MRZ release in relation to the less lipophilic surfactant CTAB. Supplementary Table S3 shows the results of a kinetic examination of the release patterns of all 22 formulations in comparison to the drug suspension. Every LPX formulation followed the Higuchi diffusion pattern, which confirmed that the LPX nano formulation sustained MRZ diffusion.

#### 2.2.4. Ex Vivo Drug Permeation Study

Supplementary Table S2 illustrates the recorded penetration parameters for the MRZ-LPX formulations. A significant difference was seen in the efflux of the treated nasal mucosa for all formulations and the MRZ suspension. After 24 h, the MRZ diffusion per unit area (Q24) in the nasal tissue of camel mucosa varied between 327.5 ± 20.2 and 445.63 ± 13.82 μg/cm^2^ for all MRZ-LPX formulations. However, the Q24 of the MRZ suspension containing the same amount of drug was 251.81 ± 11.35 (μg/cm^2^). Supplementary Figure S2 represents the permeation profile for all formulations compared with the drug suspension. The greatest fit for the response variable Y4 (Q24 µg/cm^2^) was determined to be the quadratic model based on the F value (130.44). Equation (4) uses coded values to represent the consequence of the uncorrelated variables on Q24:

(4) $${Q24}^{-2.43}=+5.541E-007+(2.001E-007)A+(8.275E-008)B-(1.118E-007)C-(6.984E-008)AC+(8.176E-008)A^{2}\;$$

The Q24 values declined when the lipid content escalated due to reduced bilayer pliability, heightened stiffness of vesicles, and augmented medium viscosity [17,24]. This could result in sluggish medication diffusion into the dissolving solution. Conversely, the Q24 values exhibited an increase when the surfactant concentration was elevated; this phenomenon might be attributed to the creation of diminutive vesicles, thus resulting in an extra surface area. Moreover, the use of SAA for lipid solubilization resulted in elevated diffusion values from LPXs [35].

As a matter of fact, it is unlikely for cationic nanovesicles with a positive charge to permeate the nasal mucosa. Instead, they tend to cling to the negatively charged mucus. Hence, transmucosal medication administration probably involves a passive diffusion mechanism that is affected by the extended retention period [34]. 

In broad terms, LPX formulations containing DDAB demonstrated superior permeation assets in comparison to those containing CTAB. The results presented here align with the investigations carried out by Shah et al. [36]. Electrostatic interactions between CTAB and the negatively charged phosphate group in PL90G may be clarified. Thereby, the supply of enough unattached CTAB to electrostatically attract the negatively charged mucosa would be impaired. An interaction between DDAB and PL90G differs discernibly. As a result, a potentially greater amount of DDAB than CTAB could be available for electrostatic interaction with negatively charged mucous [37]. Figure 1d shows the impact of several autonomous factors on Q24 (µg/cm^2^) using a three-dimensional surface plot.

### 2.3. Selection of the Optimal MRZ-LPX Formula

According to the results in Supplementary Table S4, the optimum formula with a desired index of 0.6 consists of 1.21% w/v of PL90G and a PL90G: CTAB ratio of 3. The results showed a strong resemblance between the actual and anticipated values of the optimum formulation, together with low bias percentages, suggesting that the optimization procedure was trustworthy. Supplementary Figure S3 compares the permeability and MRZ release of the regular MRZ suspension with those of the enhanced formulation.

#### 2.3.1. Morphology and Physical Stability of the Optimized MRZ-LPX

**Figure 2:**
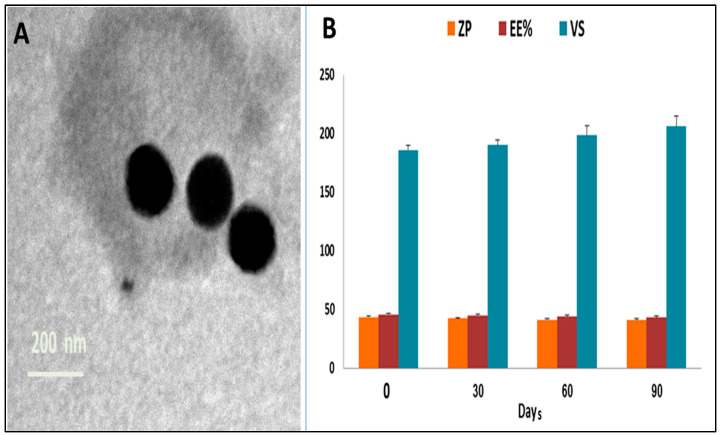
(**A**) TEM photomicrograph for the optimized MRZ nano-leciplex formulation. (**B**) Results of stability studies for the optimized MRZ nano-leciplex formulation.

The TEM images of the boosted MRZ-LPX formulation exhibited distinct vesicles with a virtually flawless spherical morphology devoid of aggregations or drug crystallization (Figure 2A). Both VS values ascertained using TEM and that via DLS were approximated. Figure 2B demonstrates that the modified MRZ-LPX formulation exhibited comparable EE%, VS, and ZP during storage for 3 months to the recently developed one. Moreover, there was no indication of sedimentation, fusion, or stratification in the optimal formulation, verifying its physical integrity.

#### 2.3.2. Z-Potential of the Tailored MRZ-LPX

ZP is a pivotal factor affecting colloids’ stability, and formulations with surface charges above +30 mV or below −30 mV are considered stable [38]. The MRZ-LPX optimized formulation’s ZP values were near +43.7 mV, indicating particles with enhanced stability in terms of electrostatic repulsion stabilization (Supplementary Figure S4C). Implementing a cationic surfactant as CTAB in the MRZ-LPX formulation was the main cause of the ZP positive value. Moreover, the ZP positive charge plays a significant influence in in vivo effects, particularly potential cellular interactions [18,39]. This positive charge can be anticipated for intranasal medication administration, since cationic vesicles interact directly with the negatively charged sialic acid moieties in the nasal mucosa [17]. This might augment adhesion to the nasal mucosal layer, thus enabling enhanced drug diffusion.

Additionally, Supplementary Figure S4D demonstrates the result of the ZP distribution curve for the CS-MRZ-LPX formulation with a result of +57.5 mV. Augmentation of the positive charge on the surface of the vesicles occurred with the addition of CS.

#### 2.3.3. Ex Vivo Mucosal Adhesion and Viscosity Examination

Mucoadhesiveness is a crucial attribute for intranasal administration as it limits the clearance for nasal formulation and enhances its contact duration. The mucoadhesiveness for the improved preparation was determined to be 5100.65 ± 27.63 dyne/cm^2^ after CS coating (CS-MRZ-LPX formulation), while the mucoadhesive force was calculated to be 3260.44 ± 22.5 dyne/cm^2^ for the MRZ-LPX formulation. Clearly, CS increased the mucoadhesion force of the formulation. The primary factor responsible for chitosan’s potent mucoadhesive effect on the nasal cavity is the presence of amine functional groups within this linear polysaccharide. Anionic substances of the nose mucus layer, such as sialic and sulfonic acids, and cationic amino compounds in chitosan (CS), interact electrostatically to improve cellular uptake and absorption by causing localized membrane instability [40,41].

A comparative analysis of the viscosity of the formulation before and after CS addition was conducted. The viscosity of MRZ-LPX was determined to have a maximum value of 396.30 ± 12.56 cp, and the viscosity of CS-MRZ-LPX was 1563.72 ± 28.4 cp. From the results, it was concluded that chitosan had a positive effect on the formulation viscosity, which was the reason why the nasal residence time and the sustained drug release were enhanced.

### 2.4. Pharmacodynamics Assessment

There is a correlation between neurotransmitter deficiency, motor dysfunction, and the persistence of depression [42]. A substantial increase in behavioral activity was observed upon the administration of the tailored CS-MRZ-LPX formula. The phenomenon was ascribed to the restoration of neurotransmitter levels in the cerebral cortex and hypothalamus induced by MRZ. When the neurotransmitter levels were enhanced, rats’ climbing and swimming time increased, and other behavioral activities improved.

#### 2.4.1. The Forced Swimming Test (FST)

**Figure 3:**
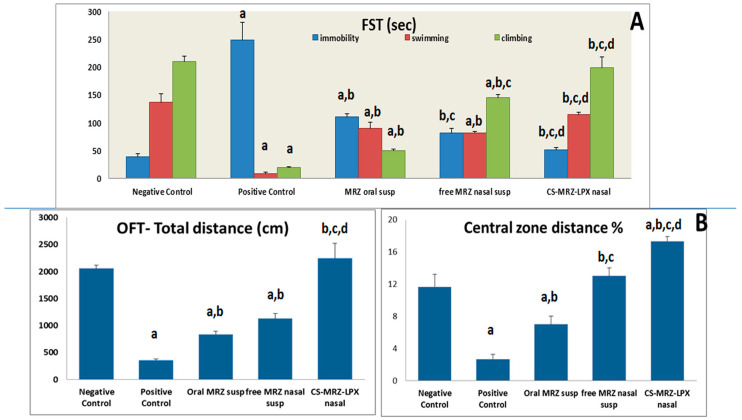
(**A**) FST behavioral analysis (immobility, swimming, and climbing) for MRZ oral suspension, the MRZ-nasal suspension, and CS-MRZ-LPX intranasal nano formulation in comparison to normal control and positive depressed rats. (**B**) OFT behavioral analysis of the total distance (cm) and the central zone (%) traversed by MRZ oral suspension and the CS-MRZ-LPX nasal preparation compared to negative and positive controls. ^a^ *p* < 0.05 relative to the negative control; ^b^ *p* < 0.05 relative to the positive control; ^c^ *p* < 0.05 relative to MRZ-susp (oral); ^d^ *p* < 0.05 relative to MRZ nasal suspension.

The FST is the most crucial assessment for evaluating the efficiency of antidepressant medicine. The concept behind the FST involves establishing a state of immobility and then evaluating the potential of antidepressant drugs to re-establish it [43]. A statistically significant difference (*p* < 0.05) was observed in the average duration of immobility between the two treatment groups (oral and nasal) and the regular or depressed standard group. Moreover, as illustrated in Figure 3A, the average duration of immobilization for the intranasal MRZ treatment group (50 ± 5 s) was significantly (*p* < 0.05) reduced compared to that of the oral MRZ treatment group (109.7 ± 6.67 s). The climbing and swimming times of the MRZ intranasal treatment group were superior to those of the control depressed group and the traditional MRZ oral group.

#### 2.4.2. Open Field Test (OFT)

During the OFT, rats diagnosed with depression exhibited a considerably decreased central zone distance and an overall distance traversed in comparison to rats treated by the optimized CS-MRZ-LPX formulation intranasally and standard negative control rats (*p* < 0.05) (Figure 3B).

#### 2.4.3. Tail Suspension Test

**Figure 4:**
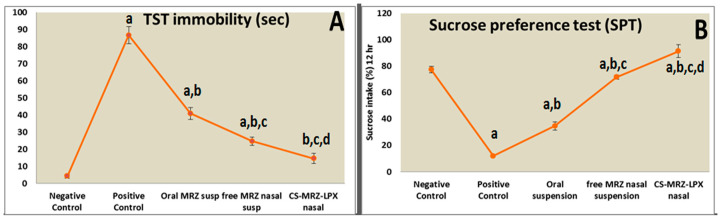
(**A**) Total time in seconds for immobility in the TST for oral and nasal preparations compared with normal control and depressed rats. (**B**) Effect of stress of food deprivation in rats (Sucrose Preference Test) within 12 h for oral and nasal preparations and the negative and positive control groups. ^a^ *p <* 0.05 relative to the negative control; ^b^ *p <* 0.05 relative to the positive control; ^c^ *p <* 0.05 relative to MRZ-susp (oral); ^d^ *p <* 0.05 relative to MRZ nasal suspension.

The results presented in Figure 4A demonstrate that the antidepressant activity of MRZ nasal dosing is superior to that of the oral dose. This is supported by a reduction in immobility duration (*p* < 0.05) for rats that received CS-MRZ-LPX intranasally (14.7 ± 2.8 s) compared to the oral suspension (41.7 ± 3.5 s) and the free MRZ nasal suspension (24.7 ± 2.3 s).

#### 2.4.4. Sucrose Preference Test (SPT)

The sucrose preference of despondent rodents was comparatively diminished in relation to the control rats (*p* < 0.05). The sucrose percentage utilized by rats that were administered MRZ orally was the lowest (34.7 ± 3.05%) in comparison to those administered with the CS-MRZ-LPX nasal treatment (91.3 ± 4.9%) (Figure 4B).

#### 2.4.5. Nasal Histopathological Examination

**Figure 5:**
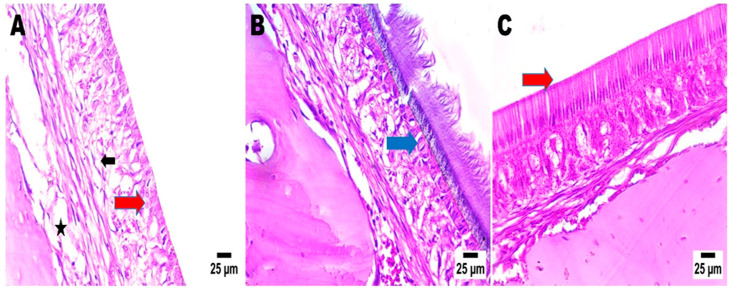
Effects of different treatments (negative control (**A**), free MRZ nasal suspension (**B**), and CS-MRZ-LPX intranasal (**C**)) on the mucosal nasal epithelium. The nasal wall in groups (**A**,**B**) had an intact epithelial lining (arrow), a submucosa with average cellularity, and average nasal cartilage. All photos were subjected to hematoxylin eosin and seen with magnification of ×400. Black arrow indicates intact sub epidermal or dermal cellularity (average cellularity). Star: no congestion in submucosal blood vessels in group (**A**). Blue arrow refers to slight congestion of blood vessels in group (**B**), which completely disappeared in group (**C**) (red arrow).

Nasal mucosa was investigated from standard negative, free MRZ nasal suspension, and intranasal CS-MRZ-LPX treatment groups. Each of the three groups’ nasal mucosa histology specimens is shown in Figure 5. The nasal epithelium in the group that received CS-MRZ-LPX nasal treatment exhibited normal morphology and displayed no indications of inflammation or assault (Figure 5C).

#### 2.4.6. Immunohistochemistry and Scoring Analysis

**Figure 6:**
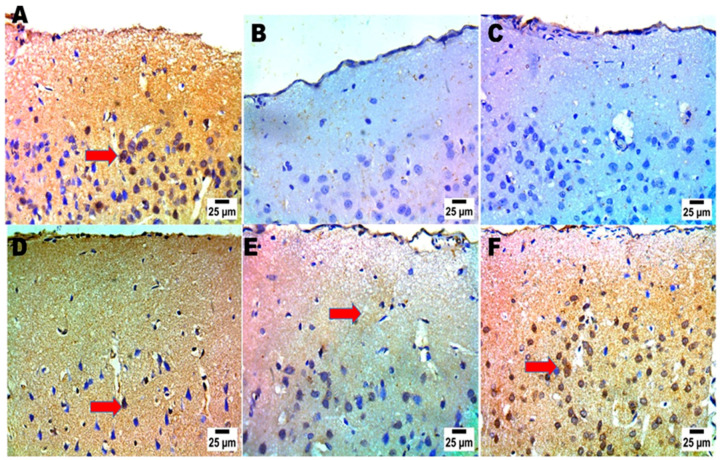
Effects of different treatments (negative control (**A**), positive control (**B**,**C**), MRZ suspension (oral) (**D**), free MRZ intranasal suspension (**E**), and intranasal CS-MRZ-LPX (**F**)) on the neurological activity and positive immune cells (%) (red arrows) in the cerebral cortex of the brain after depression induction.

**Figure 7:**
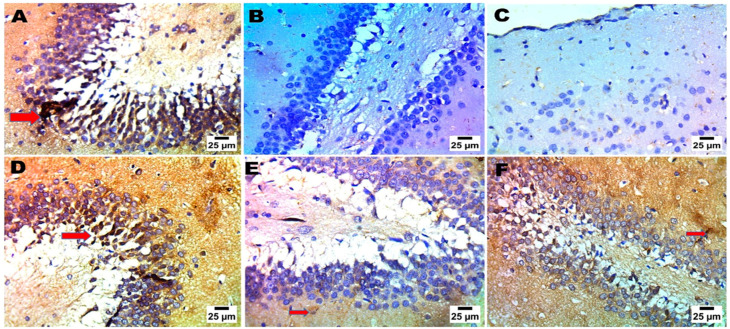
The impact of various interventions (negative control (**A**), positive control (**B**,**C**), MRZ suspension (oral) (**D**), MRZ intranasal suspension (**E**), and intranasal CS-MRZ-LPX (**F**)) on the neurological activity and percentage of positive immune cells (red arrows) in the hippocampus of the brain after depression treatment.

The current study employed immunohistochemistry to precisely determine the protein expression location of BDNF in the cerebral cortex and hippocampus, as well as to identify particular brain regions that are involved in the regulating behaviors during the FST [44]. A disruption or decreased BDNF levels can impede the plasticity of synapses, decrease the number of excitatory cells, and elevate the level of glutamate; each of these have the potential to induce depressive symptoms. The affirmative cohort had the minimal BDNF cellular tallies inside the cerebral region. It is noteworthy that the group supplied with CS-MRZ-LPX contained the highest concentration of BDNF cells in the cerebral cortex and hippocampus areas (Figure 6 and Figure 7), as well as a significant percentage of scoring (Supplementary Figure S5).

### 2.5. Pharmacokinetics Assessment

**Figure 8:**
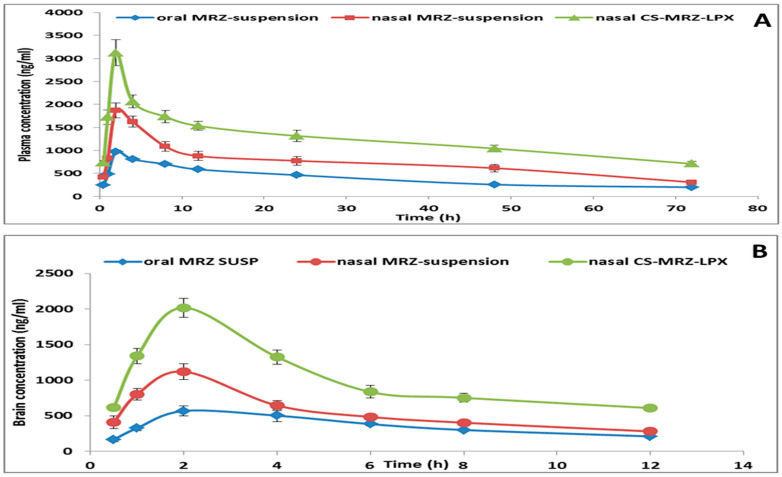
(**A**) Plasma MRZ level time profiles of MRZ oral suspension, nasal MRZ suspension, and intranasal CS-MRZ-LPX. (**B**) Brain concentration time profiles of oral MRZ suspension, nasal MRZ suspension, and intranasal CS-MRZ-LPX.

The obtained PK parameters are displayed in Table 3, and Figure 8A,B represents the MRZ’s average plasma and brain levels, respectively, in relation to the time following drug delivery for each group. Cmax, AUC^0-inf^, Ke, and MR time were substantially better in the customized intranasal CS-MRZ-LPX formulation versus the oral MRZ and intranasal MRZ suspension treatment groups (*p* < 0.05). Plasma maximum level after the intranasal CS-MRZ-LPX dosing was seen to be 3.21-fold larger than that of oral MRZ administration. When compared to oral MRZ suspension administration, intranasal administration of the CS-MRZ-LPX formulation increased the MRZ level in brain by up to 4.08 times, demonstrating the formulation’s targeted transport to the brain. The optimal intranasal formulation results in a somewhat lower brain Tmax (2 h) than the oral MRZ treatment; this difference may be attributed to a higher rate of MRZ intranasal absorption. Moreover, in the brain and plasma, the optimized CS-MRZ-LPX (intranasal) had a considerably decreased clearance rate (Table 3). Astonishingly, the nasal CS-MRZ-LPX had a relative plasma and brain bioavailability of around 370.9% and 385.65%, respectively. This implies that mucoadhesive CS-LPX is preferred for nose-to-brain delivery of MRZ [45].

Orally administered MRZ has low bioavailability since it encounters the BBB and is metabolized by the liver in the first pass. However, the intranasal route circumvents the BBB and facilitates the direct transfer of MRZ from the olfactory region of the nasal cavity into the cerebral spinal fluid and cerebral tissue, hence increasing MRZ brain biodistribution [46,47].

The following ideal features were all combined to explain the enhanced speed and degree of MRZ bioavailability utilizing the intranasal delivery of CS-MRZ-LPX: (1) Excellent permeability and solubilizing effects for MRZ are shared by CS and LPXs (carrier systems). (2) Surfactants in the nanosystem operate as penetration enhancers by lowering interfacial tension across the mucosal membrane [48]. (3) Numerous negatively charged groups can be found on the surface of cell membranes. It is anticipated that these will improve the ionic connections between positively charged LPX and enhance their nasal residence time and eventual endocytosis [49,50]. (4) The ability of CS-MRZ-LPX to shield the loaded MRZ from chemical and biological destruction in the nose permits more MRZ to reach centrally. (5) According to Mistry et al. [51], our nanoparticles’ <200 nm size makes it simple for them to be incorporated via an endocytic process through the olfactory and trigeminal nerves. (6) MRZ tight junction paracellular diffusion across epithelial cells is enhanced due to CS’s mucoadhesive characteristic, which allows nanovesicles to adhere to nasal mucosa for extended periods. Nasal epithelium cells’ negatively charged sialic acid residues or CS-specific interactions with the protein kinase C cascade are likely to be responsible [52,53].

## 3. Materials and Methods

### 3.1. Materials

As a gift sample, MASH Premiere (Cairo, Egypt) acquired Mirtazapine (MRZ), while lipoid GmbH (Nattermannallee, Germany) provided phospholipon 90G (PL90G). Sigma-Aldrich, St. Louis, MO, USA, supplied highly purified diethylene glycol monoethyl ether (Transcutol^@^ HP), chitosan (low molecular weight 15,000 Da and 75–85% degree of acetylation), cetyltrimethylammonium bromide (CTAB), and dimethyldidodecylammonium bromide (DDAB). 12–14 kDa cellophane membrane, acetonitrile, methanol, carbamazepine, and diaminobenzidine (DAB) were also purchased from Sigma-Aldrich (St. Louis, MO, USA). Rabbit BDNF polyclonal antibody (dilution: 1: 500; Novusbio, Cat. No. NB100-98682). ABC indicates Avidin Biotin-Peroxidase Combination; Vector Laboratories produces the Vectastain ABC-HRP kit. Potassium dihydrogen phosphate, disodium hydrogen phosphate and sodium chloride were purchased from El-Nasr Pharmaceutical Company, Egypt. All chemicals were of analytical grade. Double-deionized water was used throughout the research.

### 3.2. Experimental Design and Optimization

In order to fabricate and optimize MRZ-LPX formulations, central composite design, CCD (face-centered), comprising three central points, was implemented [24,54]. The independent variables encompassed the PL90G concentration (expressed as % w/v, A), the PL90G/SAA molar ratio (B), and the type of SAA (C), where A and B are numerical factors, while factor C is categorical. The values of each variable were ascertained via exploratory studies and characterized by three levels and a pair of surfactants (SAAs). Eleven iterations were carried out, eight of which included the experimental trials and three center points. Subsequently, the design was replicated for each level of C. The responses pursuant to investigation were encapsulation efficiency (Y1, EE %), LPX vesicle size (Y2, VS, nm; Y3, CMRZR %) for the cumulative MRZ released, and the total drug penetrated per square centimeter (Y4, Q24, µg/cm^2^). The experimental data were examined using the Design-Expert^®^ program, version 12.0.3.0 (Stat-Ease, Inc., Minneapolis, MN, USA), to autonomously determine the main impacts of those aspects. Finally, ANOVA was conducted to evaluate the significance of each element. The composition and constituents of the MRZ-LPX formulations created by the CCD are shown in Table 2.

#### 3.2.1. Fabrication of MRZ-LPXs

A single-step strategy was used to create LPX formulations with varied PL90G: SAA molar proportions (Table 2). Initially, PL90G and 10 mg MRZ were meticulously measured and dissolved in 0.5 mL of Transcutol HP in a sonicator water bath (Sonix TV ss-series ultrasonicator, USA) set to 70 °C until a visually transparent yellow solution with uniform consistency was achieved. Thereafter, a 9.5 mL aqueous phase comprising predetermined quantities of CTAB or DDAB at 70 °C was then added to the lipid and cyclomixed at about 1200 rpm until a homogeneous dispersion was obtained [17].

#### 3.2.2. In Vitro Exemplification of MRZ-LPXs

##### Entrapment Efficiency

Centrifugation was implemented to separate the enticed MRZ from the resultant MRZ-LPX dispersions. This method was adapted using a cooling centrifuge for three hours (SIGMA 3-30K Germany) at a speed of 20,000 rpm and a temperature of 4 °C. One milliliter of the separated LPXs from each formulation was dissolved in five milliliters of methanol for vesicle rupture. At a 289 wavelength, the MRZ concentration was determined utilizing a UV spectrophotometer (Jasco V-530, Tokyo, Japan) [17,55]. The UV spectroscopic measurement assay for MRZ was conducted according to the previously validated method [56]; the resulting linearity range of MRZ was 5–40 µg/mL, and the accuracy % recovery was 99.12 ± 0.43 with %R.S.D of 1.30%. Also, the LOQ and the LOD values were 1 µg/mL and 0.2 µg/mL, respectively. The observations were recorded in triplicate at different times. Equation (5) was applied to calculate the percentage of MRZ entrapment (EE %):

(5) $$EE\%=\left(\frac{Entrapped\;MRZ\;in\;mg}{Total\;MRZ}\right)\times 100$$

##### Determination of LPXs-VS

At 25 ± 2 °C, the polydispersity index (PDI) and the mean sizes of MRZ-LPXs were determined 3 times by employing a Zeta Sizer (Malvern Instrumentation, Malvern, UK) using the DLS method (dynamic light scattering). Prior to testing, the newly created nano-suspensions were diluted (1:10) in deionized water. The scattering angle was set at 90 degrees [57,58].

##### In Vitro Release Study of MRZ-LPXs

The MRZ release from the custom-built LPXs was assessed in triplicate utilizing a USP dissolving tester (Hanson Research, Chatsworth, USA; SR 8 Plus model) employing membrane diffusion [59]. In accordance with the calculated EE%, precise amounts of MRZ-LPX pellets (equivalent to 3 mg MRZ) were carefully dumped into glass cylinders, which were encased on one side by a dialysis membrane with a M.wt cutoff of 12,000 Da. The filled cylinders were fastened to the USP dissolving tester’s device shafts. As the release medium, 50 mL of SNES (simulated nasal electrolyte solution) with a pH of 5.5 and tween 80 (0.1% *v*/*v*) were utilized to ensure optimal sink conditions [60]. The temperature and rotational speed were adjusted to 37 ± 0.5 °C and 100 rpm, respectively. At various intervals up to 12 h, a 2 mL fraction was taken to be measured and refilled with a comparable fresh medium size to maintain a consistent volume. The cumulative MRZ released (CMRZR %) was estimated spectrophotometrically at λmax 289 (Equation (6)). The means (±SD) were then graphed against time. Likewise, release testing was conducted on the free MRZ suspension in distilled water (1.5 mg/mL, 2 mL). The obtained data were matched with zero and first-order or diffusion equations in order to examine the release kinetics of MRZ-LPXs.

(6) $$\%CMRZR=\%\;release\;at\;time\;t+\left(\frac{Sample\;volume\;withdrawn}{total\;media\;volume}\right)\times \%\;released\;previously\;(t-1)$$

where sample volume withdrawn equaled 2 mL at each interval, total media volume was 50 mL fixed throughout the assay, and %released (t − 1) is the cumulative MRZ released at the previous interval.

##### Ex Vivo Permeability Investigation of MRZ-LPXs

The camel’s nasal tissue was obtained and soaked in a solution of PBS at pH 6.4, to be employed in the permeability assessment [61]. The upper nasal concha was fixed onto a Franz diffusion cell after isolation. The temperature of the receptor compartment holding 50 mL of PBS (pH 6.4) with 0.1% (*v*/*v*) tween 80 as a permeation media was set at 37 ± 0.5 °C and the rotation speed was 100 rpm. Volumes of MRZ-LPX dispersions (equal to 3 mg MRZ) were loaded in the donor chamber. At scheduled times up to 24 h, two milliliter aliquots were aspirated from the receiver part, and it was supplied by equivalent amounts of fresh media. For each LPX formulation, the MRZ amount permeated was measured spectrophotometrically. Then Q24 (µg/cm^2^) was calculated according to Equation (7) [11].

(7) $$Cumulative\;amount\;permeated=Vol1\times Ct+\left[Vol2(\sum Ct+\cdots +Ct-1)\right]$$

where Vol1 denotes the volume of the receptor compartment, the volume obtained at every point is denoted by Vol2 (2 mL), and sample concentration at time t by Ct. For each dispersion, the diffusion attributes Q24 (µg/cm^2^), lag time (min), permeability coefficient (Kp, cm/h), and drug flow (Jss, µg/cm^2^.h) were calculated alongside the control MRZ suspension. The enhancement index (EI) was also calculated using the following equation (Equation (8)) [62]:

(8) $$EI=\frac{Kp\;of\;the\;MRZ-LPX\;formulation}{Kp\;of\;the\;control\;MRZ\;suspension}$$

#### 3.2.3. Optimization and Characterization of the Tailored MRZ-LPX Formulation

The optimal formula was derived utilizing constraints on EE%, CMRZR%, and Q24 to ensure that MRZ-LPXs reached their maximum values and on VS to provide the minimum value determined by the desirability execution. A recommended choice was made for the solution whose desirability value was near one. Following this, the program refined the expected dependent responses and the reliability of the selected optimal formulation variables was detected by customizing and evaluating the formulation in triplicate.

##### Transmission Electron Microscopy (TEM)

The morphology of MRZ-LPX optimal formulation was evaluated using an electron microscope with transmission (JEM-1400, Jeol, Tokyo, Japan). On a copper grid, a single drop of the ideal LPX preparation was applied and any extra was eliminated. Then, an aqueous solution containing negative staining (2% w/v phosphotungstic acid) was added. After being air-dried, the samples were examined at 80 Kv in a TEM [61,63].

##### Physical Stability Study of MRZ-LPX

The optimum MRZ-LPX preparation was tested for stability by keeping it in a glass vial at 4 °C for three months. Samples from the optimal formulation were obtained at storage durations of 1, 2, and 3 months after fabrication. The obtained samples were tested for EE%, VS, and Z-potential, with tests repeated thrice [64].

##### Zeta Potential Measurement

Applying a Malvern Zeta Sizer (Malvern, UK), the Z-potential of the ideal nano cationic dispersion was determined, and the mean of three measures (*n* = 3) was computed. After applying an electrical field, the velocity of vesicles through a liquid was measured using an electrophoresis-based approach [65].

#### 3.2.4. Formulation of CS Grafted MRZ-LPX

The chitosan-coated LPX formulation of MRZ (CS-MRZ-LPX) was prepared using the same one-step method described previously for fabrication of MRZ-LPX except for one point. The difference in formulation preparation was in the aqueous phase. A quantity of 9.5 mL of acidic distilled water containing 0.5% *v*/*v* acetic acid was used to dissolve CTAB and 35 mg of CS. Then, this aqueous phase was added and cyclomixed with 0.5 mL of PL90G and MRZ solution in transcutol HP. The concentration of CS utilized was determined according to trials and review studies [10,66].

##### Ex Vivo Mucoadhesion Study and Viscosity Assessment

A modified physical balancing technique assessed the enhanced CS-MRZ-LPX formulation’s bioadhesive strength. The strength needed to detach the preparation from the nose mucosal membrane was estimated by a cm^2^ segment of newly dissected camel nasal tissue. A milliliter of the optimal formulation was placed on the first disc, which was set on a height-adaptable pan. For instance, a different slide was linked to the balance with the nasal mucosa anchored in an upturned point. Both slides, including the formulation between them, were held close together for 2 min. The load kept rising at the balance’s other edge; eventually, both slides separated [67]. The adhesive force (dyne/cm^2^) was tested three times using the minimum load that could break off the two plates, as follows:

(9) $$Mucoadhesive\;strength\left(Dynes/{cm}^{2}\right)=\frac{m\times g}{A}$$

where m in grams signifies the weight required for slide separation, g is the acceleration of gravity (980 cm/s^2^), and the surface area of the exposed nasal is A.

The viscosity of the optimized MRZ-LPX formulation prior to and subsequent to CS coating was investigated using a cone and plate viscometer (Brookfield DV-III ULTRA, USA). The investigation of the viscosity of formulations was measured at shear rates ranging from 20 to 200 (s − 1).

#### 3.2.5. Pharmacodynamics Study

##### Animals

The antidepressant competence of the grafted CS-MRZ-LPX formula was assessed in a collection of 30 adult male Wistar rats weighing between 160 and 180 g. The rats were partitioned into five distinct groups. Before depression induction or undergoing experimental procedures, the rats were acclimated to a regular rodent diet for a period of 7 days as soon as they arrived. They were also provided with a consistent supply of fresh drinking water.

##### Depression Induction and Experimental Procedure

The forced swim test (FST) was used in this animal inquiry to induce despondency. Kaur et al. conducted a similar study where a cohort of rats received regular swimming lessons for seven days [43]. Except for the control group, all the animals were immersed in water maintained at a temperature of 25 ± 2 °C inside a cylindrical container. They swam for 15 min on the initial day and then for an additional 5 min during each swim session, during both the day and at night. After each swim, the rodents were removed from the water, dried, and reintroduced into their enclosures. Within G1, the rats demonstrated no evidence of depression induction, therefore functioning as the group for the negative control. Conversely, as a positive monitoring cohort in G2, the rats received isotonic saline solution (0.5 mL), which served for distressed animals. G3 was given MRZ suspension (orally) in clean water (2.5 mg/mL; 15 mg/kg) [68]; G4 received intranasal administration of 100 μL (in each nostril) of MRZ suspension (15 mg/kg); and G5 was administered an equivalent dosage to group 4, both in terms of manner and quantity, except for the modified CS-MRZ-LPX formulation. The intranasal administration was conducted using a tiny pipette attached, whilst animals were firmly restrained in a tilted posture to guarantee precise application. Further behavioral examinations were deftly established.

##### Forced Swimming Test (FST): Immobility, Bathing, and Rambling

During the FST, the rodents were confined inside a vessel overflowing with water. After a 10-min drug administration, the phases of apathy, climbing, and floating were astutely observed. After ceasing its battle and surrendering, the rat appeared motionless and floated in the water [69].

##### Open Field Test (OFT)

The rodents were given a generous 6-min period to freely explore their designated areas subsequent to their placement. Within the confines of a 100 × 50 cm obsidian cuboid cage, rats acclimated to their immediate environment during the first thirty seconds. During the test period, the length spent in the middle of the OFT box and the total distance covered were estimated using camera video footage [70].

##### Tail Suspension Test (TST)

For the purpose of suspending rodents by their tails from 50 cm high rods, adhesive tape was employed. A mounting spigot impeded the rat’s ability to ascend its tail. After thirty minutes of drug dosage, the duration of immobility and lack of attempts to escape for 6 min in each rat were monitored and assessed [71].

##### Sucrose Preference Test (SPT)

A diminished preference for sweet food in the sucrose-based predilection test indicates despair, a condition that may be mended with antidepressant treatment. During the experimental period spanning from day one to day four, the rodents were provided with a variety of dietary items. For the first two days, the rats received 2 bottles of purified water, two bottles of sucrose (1%), and one bottle of clean water and one bottle of sucrose (1%) on the third and fourth days, respectively [70]. After 12 h of food and water deprivation, each rat was administered 200 mL of a solution of clean water and 1% sucrose. The entire amount was calculated, and the preference for sucrose was calculated using the computation (Equation (10)):

(10) $$SP\left(\%\right)=\left[\frac{consumption\;of\;sucrose\;solution\left(g\right)}{total\;consumption\left(g\right)}\right]\times 100$$

##### Investigations of Histopathology and Tolerability

Nasal mucosa histopathological inspections were executed to ascertain safety for the nasal preparation and exclude any possibility of complications [72]. Tissues were procured humanely from deceased animals in the negative control, nasal MRZ suspension, and intranasal CS-MRZ-LPX. The rodents received an intraperitoneal injection of 0.1 mL/100 g body weight xylazine (5 mg/kg) and ketamine (90 mg/kg) in a 1:1 mixture. Following surgical removal, demineralization, and preservation in a 10% buffered formalin solution, the mucosa was prepared for additional examination and sectioning. After hematoxylin and eosin (H&E) staining, the samples were scrutinized using a light microscope [73,74].

##### Brain-Derived Neurotropic Factor (BDNF) Immunohistochemical Investigation and Statistical Analysis

After completing atmospheric evaporation at 25 °C for a single night, the brain segments were submerged in 4% paraformaldehyde for 30 min. Post-fixation, the sections underwent two rinses before being incubated at 25 °C for a day. The paraffin slices were mounted onto positively charged slides using the avidin biotin-peroxidase combination (ABC). Chemicals from the ABC technique were introduced into sections from each group after antibody incubation. The marker expression was peroxidase labeled and DAB stained. The IHC-stained slices were examined with an Olympus BX-53 microscope. The results shown in the J 1.53t image, Wayne Rasband and coworkers, USA, National Institutes of Health, are scored using response area% in ten microscopic fields. Experiments conducted in triplicate are displayed as mean ± SD. The behavioral research data were evaluated using a one-way ANOVA and LSD post hoc test. SPSS 20 was utilized to conduct statistical analysis in this study. A *p*-value less than 0.05 was deemed to be empirically significant [75].

#### 3.2.6. Plasma and Brain Bioavailability Study

##### Animals

PK attributes of both oral and nasal formulations were investigated in 72 male Wistar rats weighing an average of 200–250 g. There were three groups of twenty-four rats each. After fasting overnight, the rats were given a dosage and confined for sampling.

##### Administration of MRZ to Rats and the Study Procedures

MRZ’s oral and intranasal formulations were supplied at a consistent dosage of 15 mg/kg body weight to evaluate the PK parameters [68,76]. The MRZ suspension was administered orally to the initial cohort. The MRZ suspension and the enhanced CS-MRZ-LPX formulation were administered intranasally to the second and third groups, respectively. The PK investigation ran in the brain for 12 h and in the plasma for 72 h to determine an explicit elimination phase of MRZ, which was necessary for evaluating multiple PK parameters.

Hematological specimens were obtained from anesthetized rats’ retro-orbital punctures in microcentrifuge tubes containing heparin (*n* = 3 per time point). The plasma was separated immediately after 20 min of centrifugation at a velocity of 2000 rpm. Brain tissues were extracted at the culmination at each sample interval. Humanely, rats were executed with the submission to an excessive quantity of diethyl ether inside a hermetic glass receptacle; the dissected cerebral hemispheres were immediately washed, quantified, and standardized in a 1:4 (w/v) solution of physiological saline. In homogenizer (Fisher Scientific, Germany), the homogenization process required 5 min at a speed of 20,000 rpm [46,77]. Serum and brain tissue homogenates were kept at −20 °C until the MRZ concentration was determined.

##### Sample HPLC Analysis

All stored samples were separated via liquid extraction. A quantity of 500 µL of each specimen received 0.5 mL of carbamazepine (internal standard, 1000 ng/mL) before extracting MRZ with 5 mL acetonitrile [31]. The acetonitrile–drug solutions were centrifuged at 6000 rpm for 10 min. The top layer evaporated after being delicately gathered and put into a new tube. Before transferring to the injection vials, the dried tube portion was dissolved in 500 µL acetonitrile. A 20 µL aliquot of the reconstituted extraction was injected into the HPLC apparatus for MRZ measurement. A standard plasma MRZ calibration curve was constructed from 100 to 1500 ng/mL [78]. HPLC analysis was assessed based on a previously validated technique in accordance with ICH recommendations prior to the pharmacokinetic experiments [3]. The samples were evaluated using a Waters Alliance 2695 HPLC system with Waters 2996 PDA. Rat plasma samples were isocratically eluted into Kromasil C18 (5 m, 4.6 × 150 mm) using 60:40 phosphate buffer (pH 3.9) to acetonitrile. The flow was 1.5 mL/min at room temperature. A photodiode array detector determined the drug’s wavelength at λmax 289 nm [79].

##### Pharmacokinetic and Statistical Analysis

The PK parameters were assessed utilizing the non-compartmental model WinNonlin (version 1.5, Scientific Consultants, Inc., Rockville, MD, USA). By graphing the average MRZ concentrations in brain and plasma against hours after nasal and oral administration, the maximum levels (Cmax) and time to attain them (Tmax) were estimated. The software calculated the half-life (time to achieve half of the drug plasma level) and mean residence time (MRT). The area under the curve from 0 to t h was calculated using the trapezoidal technique. Nasal relative bioavailability (Frel) vs. MRZ oral suspension was calculated implementing the following equation:

(11) $$Frel=\frac{AUC\;(CS-MRZ-LPX\;intranasal)}{AUC\;(oral\;MRZ\;suspension)}*100$$

PK characteristics were compared between the nasal CS-MRZ-LPX formulation, oral MRZ suspension, and free MRZ nasal suspension using Student’s *t*-test. Statistical significance was established at *p* < 0.05, indicating significant differences. The in vivo study used standard deviation (SD) from a minimum of three points to represent all data.

## 4. Conclusions

Various MRZ-LPX preparations were developed and adapted applying CCD. The produced formulations comprised MRZ with a rising EE%, adequate size, and improved penetration over the nasal mucosa. The infringement of liver initial-pass degradation, overcoming the BB barrier, and MRZ passing through the olfactory and trigeminal neural cells were the aspects responsible for the 3.85-fold spike in relative brain availability of MRZ using the intranasal CS-MRZ-LPX formulation compared to MRZ oral suspension. As a consequence, MRZ nose-to-brain delivery may be a viable option for MRZ therapy, with the additional benefits of ease of administration, increased patient adherence, prompt onset of action, low dosage, and reduced systemic absorption. Moreover, behavioral investigations demonstrate that nasal delivery has a superior antidepressant impact over oral dosing and a significant improvement in PK parameters. Immunohistopathological investigations showed the high efficacy and brain delivery of the tailored CS-MRZ-LPX formulation. In summary, CS-MRZ-LPX could potentially be an attractive medication delivery approach for managing depression in a safe, consistent, and perpetual way.

## Figures and Tables

**Table 1:** The CCD factors with their levels and the responses implemented in MRZ-LPX formulations.

Factor Code	Factor Name	Factor Level			Response Code	Response Name	Unit
		−1	0	+1			
					Y_1_	Entrapment efficiency	(EE %)
**A**	PL90G conc (%w/v)	1	1.25	1.5	Y_2_	leciplex vesicle size (VS)	(nm)
**B**	PL90G:SAA (ratio)	3	4	5	Y_3_	Amount MRZ released after 12 h (CMRZR)	%
**C**	SAA type	CTAB		DDAB	Y_4_	Q_24_^*^	(µg/cm^2^)

Q_24_^*^ = Cumulative amount of drug permeated after 24 h.

**Table 2:** The four CCD reactions and responses of different MRZ-LPX formulations.

Run	PL90G (%w/v)	PL90G:SAA (Ratio)	SAA Type	Y_1_ (%) ± SD	Y_2_ (nm) ± SD	Y_3_ (%) ± SD	Y_4_ (µg/cm²) ± SD
**F1**	1	3	CTAB	33.10 ± 2.55	124.20 ± 7.69	82.93 ± 2.87	445.63 ± 13.82
**F2**	1	4	CTAB	36.87 ± 2.44	146.30 ± 10.90	77.03 ± 2.05	402.87 ± 11.38
**F3**	1	5	CTAB	44.40 ± 3.12	160.73 ± 8.59	72.70 ± 2.16	369.80 ± 14.22
**F4**	1.25	3	CTAB	51.53 ± 1.46	198.27 ± 9.60	76.41 ± 1.64	378.47 ± 12.14
F5 *	1.25	4	CTAB	55.14 ± 2.74	252.20 ± 3.84	71.36 ± 1.80	352.90 ± 9.12
F6 *	1.25	4	CTAB	56.67 ± 2.65	244.90 ± 5.82	71.13 ± 1.63	340.83 ± 10.03
F7 *	1.25	4	CTAB	57.97 ± 1.96	258.60 ± 6.70	70.79 ± 2.64	346.36 ± 7.91
**F8**	1.25	5	CTAB	66.70 ± 1.35	287.57 ± 6.47	65.60 ± 4.48	334.60 ± 8.09
**F9**	1.5	3	CTAB	62.37 ± 2.71	316.17 ± 8.06	69.53 ± 1.52	302.70 ± 10.84
**F10**	1.5	4	CTAB	64.93 ± 1.93	342.50 ± 10.56	64.17 ± 2.96	292.23 ± 12.08
**F11**	1.5	5	CTAB	77.33 ± 1.76	364.56 ± 12.27	61.28 ± 3.81	286.17 ± 11.91
**F12**	1	3	DDAB	52.90 ± 3.15	303.33 ± 8.32	73.14 ± 1.04	460.46 ± 16.45
**F13**	1	4	DDAB	56.33 ± 2.52	334.57 ± 9.35	68.44 ± 1.85	437.16 ± 13.91
**F14**	1	5	DDAB	68.53 ± 2.21	398.30 ± 7.30	65.53 ± 2.80	416.52 ± 15.33
**F15**	1.25	3	DDAB	59.50 ± 1.63	363.96 ± 9.51	67.77 ± 2.66	440.77 ± 11.99
F16 *	1.25	4	DDAB	70.53 ± 3.3	391.73 ± 12.75	63.78 ± 1.48	408.63 ± 12.74
F17 *	1.25	4	DDAB	68.80 ± 2.41	384.40 ± 16.03	62.26 ± 3.59	414.96 ± 10.48
F18 *	1.25	4	DDAB	69.40 ± 2.10	378.66 ± 13.32	62.86 ± 2.34	410.40 ± 9.77
**F19**	1.25	5	DDAB	78.15 ± 2.89	427.70 ± 7.51	58.58 ± 2.04	398.26 ± 15.94
**F20**	1.5	3	DDAB	72.81 ± 3.69	363.80 ± 7.96	60.47 ± 3.17	387.30 ± 11.85
**F21**	1.5	4	DDAB	80.30 ± 2.91	448.30 ± 9.71	57.24 ± 2.99	352.50 ± 9.84
**F22**	1.5	5	DDAB	86.43 ± 3.15	483.40 ± 9.10	55.91 ± 2.48	327.50 ± 20.20

SD (standard deviation of *n* = 3). All formulations contain 10 mg MRZ, * indicates center points of the design.

**Table 3:** Pharmacokinetic parameters for the MRZ oral suspension, intranasal MRZ suspension, and intranasal CS-MRZ-LPX optimized formulation.

Pharmacokinetics Parameter	Oral MRZ Suspension		Intranasal MRZ Suspension		Intranasal CS-MRZ-LPX	
	Plasma	Brain	Plasma	Brain	Plasma	Brain
**C_max_ (ng/mL)**	972.84 ± 34.28	494.94 ± 34.21	1869.56 ± 132.82 (a)	1121.79 ± 108.72 (a)	3123.036 ± 281.57 (a,b)	2021.198 ± 133.24 (a,b)
**T_max_ (h)**	2	2.67 ± 0.94	2	2	2	2
**T_1/2_ (h)**	35.01 ± 1.86	5.82 ± 0.27	39.73 ± 1.722 (a)	7.59 ± 0.141	54.19 ± 1.28 (a,b)	14.2 ± 3.31 (a,b)
**Kel**	0.0198 ± 0.001	0.1193 ± 0.0054	0.0175 ± 0.0007 (a)	0.0913 ± 0.0017 (a)	0.0128 ± 0.0003 (a,b)	0.0511 ± 0.010 (a,b)
**MRT (h)**	51.47 ± 2.32	8.92 ± 1.20	53.48 ± 1.89	10.63 ± 0.42	75.81 ± 1.42 (a,b)	18.82 ± 4.3 (a,b)
**AUC_(0-t)_ (ng.h/mL)**	28,813.4 ± 1406.97	4278.466 ± 127.72	52,274.84 ± 4333.01 (a)	6518.425 ± 219.87 (a)	88,954.4 ± 6980.9 (a,b)	12,145.2 ± 457.11 (a,b)
**AUC _(0-∞)_ (ng.h/mL)**	38,955.64 ± 2051.04	6328.87 ± 266.57	69,950.22 ± 5507.48 (a)	9596.36 ± 508.31 (a)	144,514 ± 12,348.7 (a,b)	24,407.4 ± 2384.57 (a,b)
**% Relative bioavailability (F_rel_)**			179.5	151.6	370.9	385.6

T_max_: Time to reach cmax; C_max_: Maximum plasma concentration; MRT: Mean residence time; AUC_(0-t)_: Area under the serum concentration–time curve; ^a^ *p* < 0.05 compared to the MRZ oral suspension group; ^b^ *p* < 0.05 relative to the free MRZ suspension nasal group.

## Data Availability

Data is contained within the article or Supplementary Materials.

## Supplementary Materials

The following supporting information can be downloaded at: https://www.mdpi.com/article/10.3390/ph18010046/s1, Figure S1: The release patterns for all 22 MRZ-LPXs formulations compared to MRZ suspension; Figure S2: Cumulative MRZ permeated per unit area after 24 h (Q24 µg/cm^2^) of all the MRZ-LPXs formulations compared to MRZ suspension; Figure S3: the release (A) and permeation (B) curves for the optimized MRZ-LPX formulation compared to CS-MRZ-LPX and MRZ free suspension; Figure S4: (A) size distribution curve of the optimized MRZ-LPX formulation, (B) size distribution curve of CS-MRZ-LPX formulation, (C) Zeta potential curve for the optimized MRZ-LPX formulation, and (D) Zeta potential curve for CS-MRZ-LPX formulation; Figure S5: Effects of different treatments (Negative control, Positive control, MRZ-susp (oral), Free MRZ susp (nasal), and CS-MRZ-LPX (nasal) on the neurological activity and positive immune cells (%); Table S1: Regression analysis results for Y_1_, Y_2_, Y_3_ and Y_4_ responses; Table S2: permeation different variables for all the MRZ-LPXs formulations; Table S3: kinetic analysis of the MRZ release for different MRZ-LPXs formulations together with MRZ suspension; Table S4: The anticipated and experimental results for the four responses of the MRZ-LPX optimized formulation.
