# Supplementary material for: Nose-to-Brain Delivery of Chitosan-Grafted Leciplexes for Promoting the Bioavailability and Antidepressant Efficacy of Mirtazapine: In Vitro Assessment and Animal Studies
Source: Pharmaceuticals (Basel). 2025 Jan 3;18(1):46. doi: 10.3390/ph18010046 (PMC11768278; doi:10.3390/ph18010046)
Supplement: Supplementary file 1 [file pharmaceuticals-18-00046-s001.zip › pharmaceuticals-3333339-supplementary.pdf]

Supplementary Table S1: Regression analysis results for Y_1, Y_2, Y_3 and Y_4 responses

Response	Model	Adequate precision	R^2	Adjusted R^2	Predicted R^2	SD	% CV	P-value
**Y_1**	Quadratic	36.679	0.9820	0.9709	0.9391	2.32	3.73	<0.0001
**Y_2**	2FI	49.63	0.9878	0.9829	0.9678	12.9	4.07	<0.0001
**Y_3**	2FI	65.706	0.9913	0.9878	0.9751	0.76	1.13	<0.0001
**Y_4**	Quadratic	41.294	0.9877	0.9801	0.9376	2.988E-008	5.06	<0.0001

*R^2: coefficient of determination; SD: standard deviation; CV: coefficient of variation.

Supplementary Table S 2: permeation different variables of the MRZ-LPXs formulations

formula nom	Q24(µg/cm2)	flux(Jss) (µg/cm^2.h)	Kp (cm/h)	lag time(min)	Enhance index	PDI
**F1**	445.63±13.82	36.51±3.73	0.0365±0.0031	16.37±0.98	2.27	0.469±0.0402
**F2**	402.87±11.38	30.50±4.8	0.03050±0.0043	17.25±1.03	1.89	0.436 ±0.0842
**F3**	369.8±14.22	27.99±2.84	0.02799±0.0015	25.13±1.15	1.74	0. 431±0.0513
**F4**	378.47±12.14	29.34±2.09	0.02934±0.0028	22.98±2.65	1.82	0. 553±0.0780
**F5***	352.9±9.12	25.69±1.57	0.02569±0.0018	29.87±2.49	1.59	0.502±0.0621
**F6***	340.83±10.03	21.27±1.24	0.02127±0.0009	31.00±3.50	1.32	0.528 ±0.009
**F7***	346.36±7.91	22.75±2.28	0.02275±0.002	27.16±3.14	1.41	0.581 ±0.0395
**F8**	334.6±8.09	23.91±3.03	0.02391±0.0013	19.51±2.94	1.48	0.541 ±0.0583
**F9**	302.7±10.84	22.07±3.60	0.02207±0.0008	24.09±3.06	1.37	0.511 ±0.0937
**F10**	292.23±12.08	20.20±1.17	0.02020±0.0010	28.68±2.61	1.25	0.548 ±0.0814
**F11**	286.17±11.91	19.11±1.44	0.01911±0.0005	33.23±3.28	1.19	0.589±0.0722
**F12**	460.46±16.45	33.84±4.62	0.03384±0.0049	12.01±1.32	2.09	0.692 ±0.0911
**F13**	437.16±13.91	31.19±3.87	0.0312±0.0006	23.26±1.89	1.94	0.651 ±0.0645
**F14**	416.52±15.33	28.08±4.55	0.02808±0.0014	28.05±2.76	1.74	0.636 ±0.099
**F15**	440.77±11.99	32.74±3.24	0.03274±0.0042	20.07±1.47	2.03	0.668±0.083
**F16***	408.63±12.74	26.42±2.88	0.02642±0.0027	14.39±1.90	1.64	0.694±0.064
**F17***	414.96±10.48	36.40±4.71	0.03640±0.0058	15.93±1.26	2.26	0.627±0.00851
**F18***	410.4±9.77	28.004±2.06	0.02800±0.0036	20.72±2.20	1.74	0.616±0.0289
**F19**	398.26±15.94	25.19±3.43	0.02519±0.0029	17.03±1.29	1.56	0.791±0.0375
**F20**	387.3±11.85	24.15±1.82	0.02415±0.0007	22.79±0.86	1.49	0.728±0.089
**F21**	352.5±9.84	21.23±1.01	0.02123±0.004	19.76±1.21	1.32	0.714±0.009
**F22**	327.5±20.2	18.98±1.52	0.01898±0.0009	24.72±2.67	1.18	0.703±0.026
**MRZ susp**	251.81 ±11.35	16.12±1.94	0.01612±0.0005	40.80±4.27	-	-
**MRZ-LPX optimum formula**	383.23±13.08	27.85±3.74	0.02785±0.0008	15.46±1.06	1.73	0.531±0.0693
**CS-MRZ-LPX**	352.36±12.34	24.87±2.45	0.02487±0.0048	27.66±2.48	1.54	-

SD (Standard deviation of n=3 *** indicates center points of the design**

Supplementary Table S3: Kinetic analysis of MRZ release from different MRZ-LPX formulations together with MRZ suspension

**Formulation**	**R2**			**Mechanism**
	**Zero**	**First**	**Higuchi**	
**F1**	0.885764	0.982667	**0.986264**	Diffusion
**F2**	0.89864	0.982857	**0.989143**	Diffusion
**F3**	0.911541	0.978577	**0.997403**	Diffusion
**F4**	0.901387	0.980071	**0.996691**	Diffusion
**F5**	0.91493	0.975293	**0.991446**	Diffusion
**F6**	0.913599	0.980099	**0.997769**	Diffusion
**F7**	0.924105	0.981813	**0.996714**	Diffusion
**F8**	0.928755	0.98011	**0.997163**	Diffusion
**F9**	0.929116	0.985058	**0.99684**	Diffusion
**F10**	0.936162	0.985207	**0.998626**	Diffusion
**F11**	0.944454	0.986859	**0.997944**	Diffusion
**F12**	0.916041	0.981732	**0.994691**	Diffusion
**F13**	0.915726	0.973663	**0.99523**	Diffusion
**F14**	0.917816	0.972174	**0.995313**	Diffusion
**F15**	0.920594	0.973918	**0.993485**	Diffusion
**F16**	0.928099	0.977253	**0.996291**	Diffusion
**F17**	0.929859	0.976177	**0.99669**	Diffusion
**F18**	0.931421	0.977266	**0.995346**	Diffusion
**F19**	0.939521	0.978497	**0.996407**	Diffusion
**F20**	0.93674	0.979052	**0.997221**	Diffusion
**F21**	0.947574	0.985286	**0.999572**	Diffusion
**F22**	0.95554	0.988707	**0.999348**	Diffusion
**MRZ susp**	0.977392	**0.993442**	0.982911	First
**MRZ-LPX optimum formula**	0.918583	0.985316	**0.989886**	Diffusion
**CS-MRZ-LPX**	0.921713	0.973993	**0.99209**	Diffusion

Supplementary Table S4: The anticipated and experimental results for the four responses to the optimal MRZ-LPX formulation

**Solution**	**PL90G (%w/v)**	**PL90G: SAA (ratio)**	**SAA type**	**Y_1 (%)**	**Y_2 (nm)**	**Y_3 (%)**	**Y_4 (µg/cm^2)**	**desirability**
**Predicted Value**	1.21	3	CTAB	47.79	199.46	77.28	385.57	0.6
**Experimental Value**	1.21	3	CTAB	45.86 ± 0.76	186.2 ± 3.5	76.66 ± 3.06	383.23 ± 13.08	0.6
**Bias%**	-	-	-	4.21	7.12	0.81	0.61	-

Each single value represents the average ± (SD) (n=3).
%Bias = (predicted value-Experimental value)/Experimental value

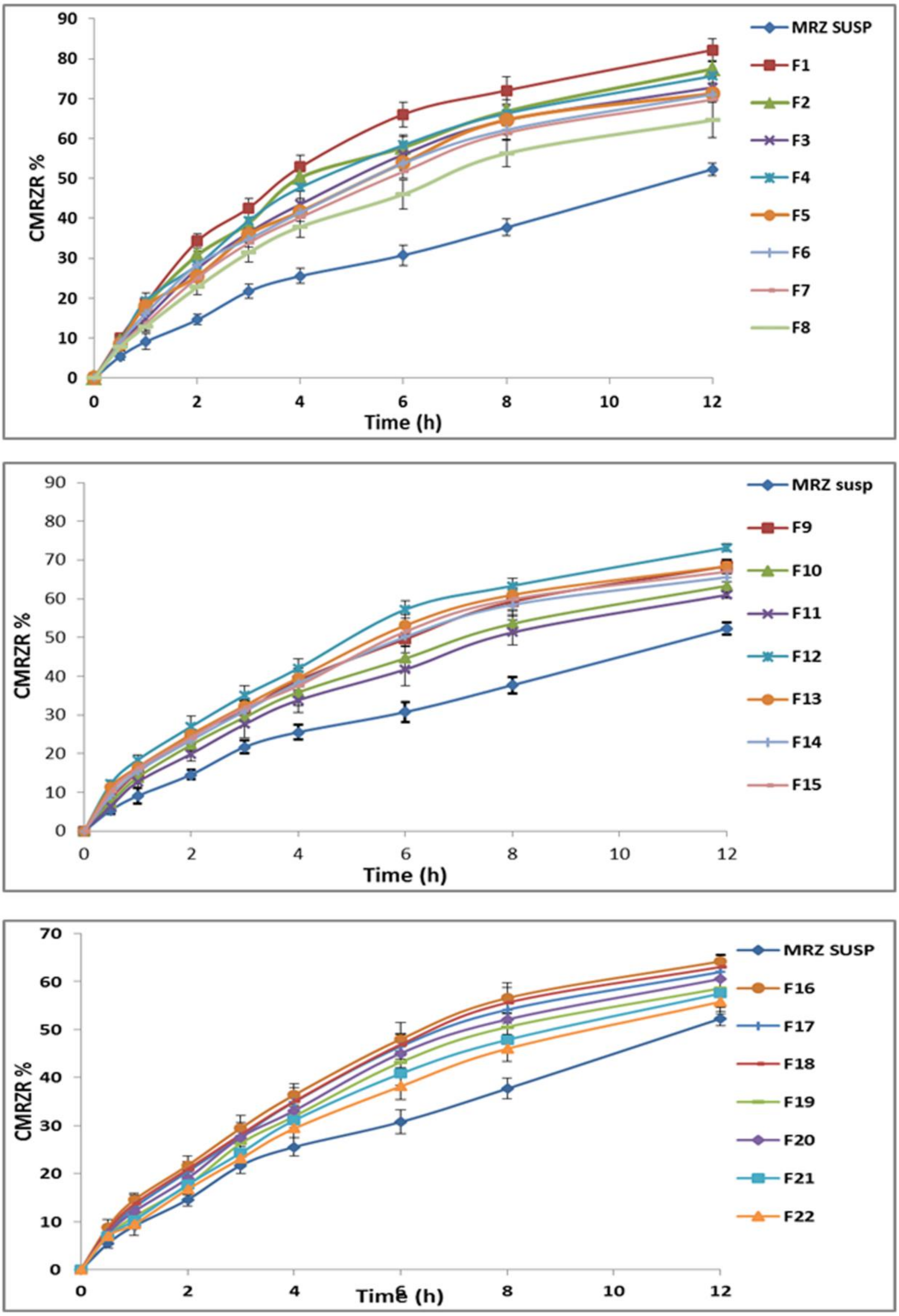

Supplementary Figure S1: The release patterns for all 22 MRZ-LPXs formulations compared to MRZ suspension

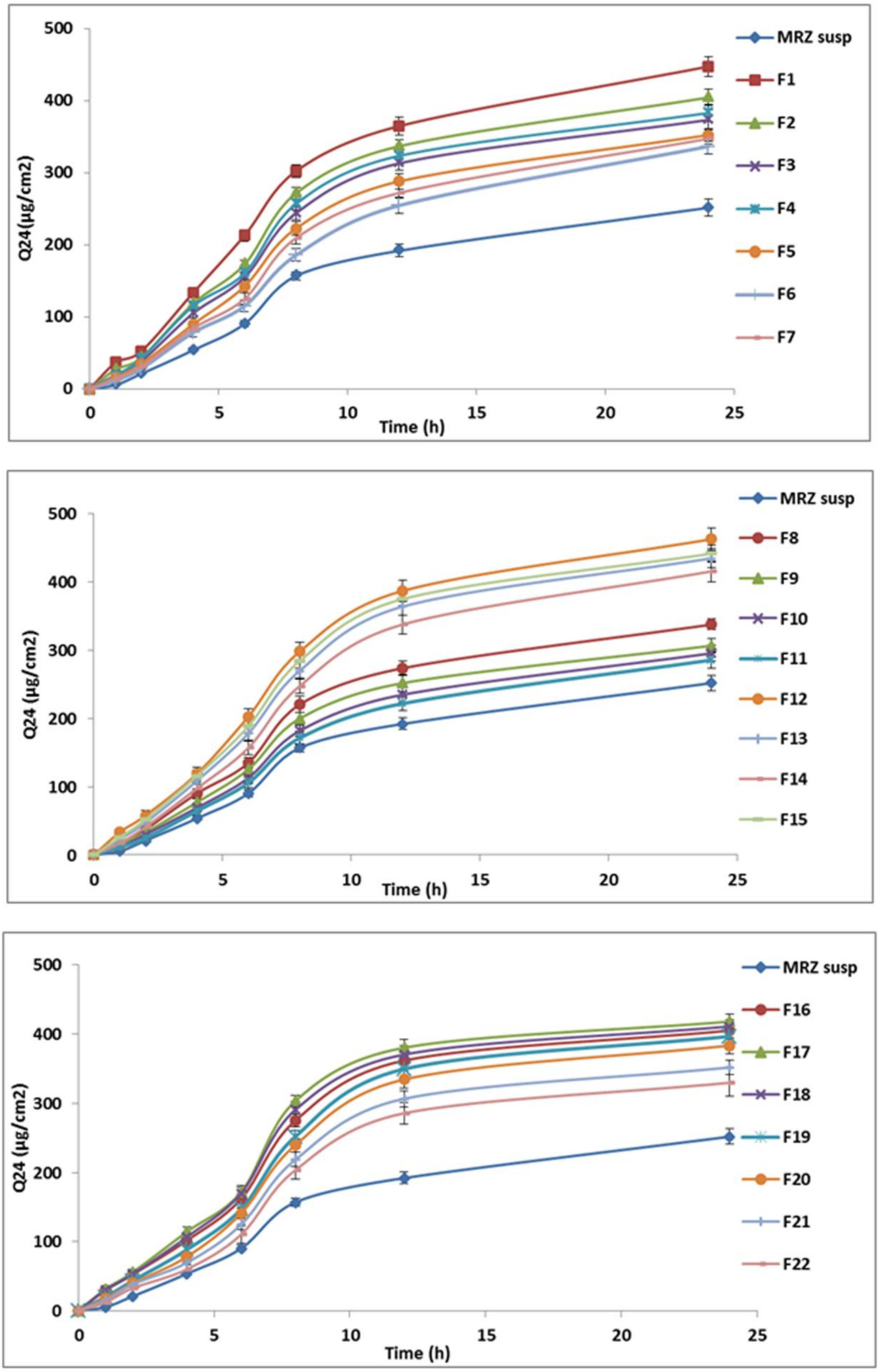

Supplementary Figure S2: The permeation patterns for all 22 MRZ-LPXs formulations compared to MRZ suspension

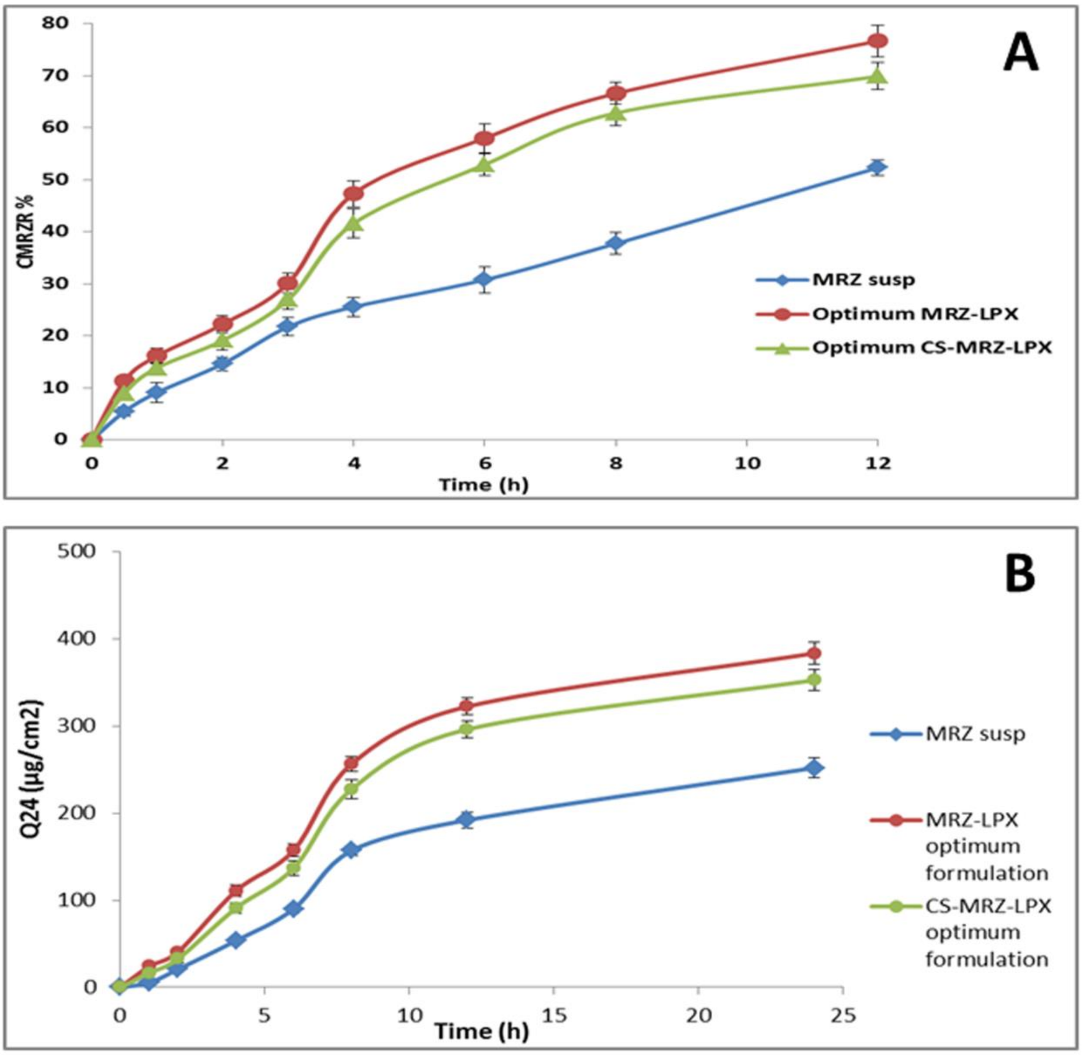

Supplementary Figure S3: release (A) and permeation (B)profiles for the optimized CS-MRZ-LPX compared to the free MRZ suspension

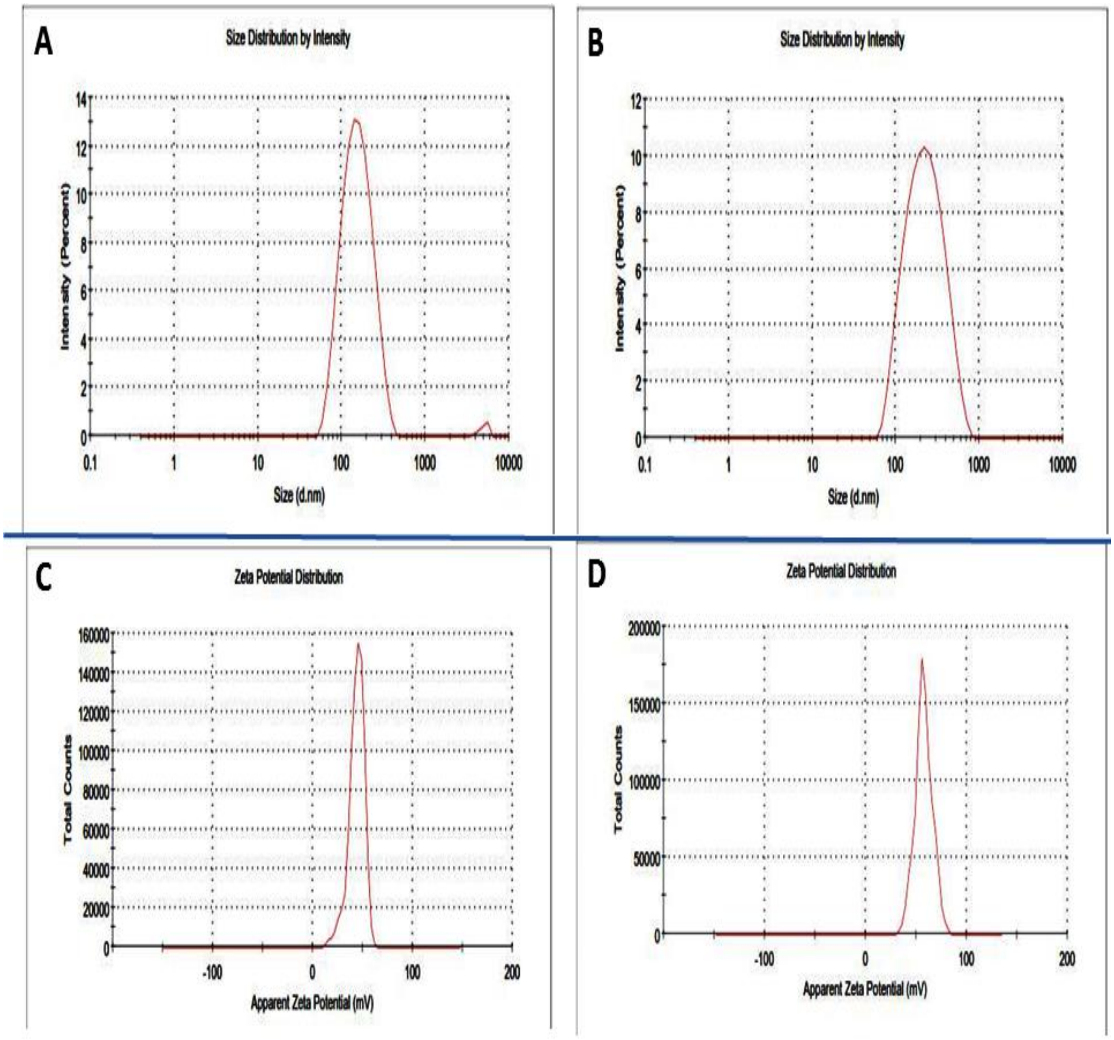

Supplementary Figure S4: (A) size distribution curve for the optimum MRZ-LPX formulation, (B) size distribution curve for the optimum CS-MRZ-LPX formulation, (C) Z-potential distribution curve of the optimized MRZ-LPX formulation, and (D) Z-potential distribution curve of the optimized CS-MRZ-LPX formulation

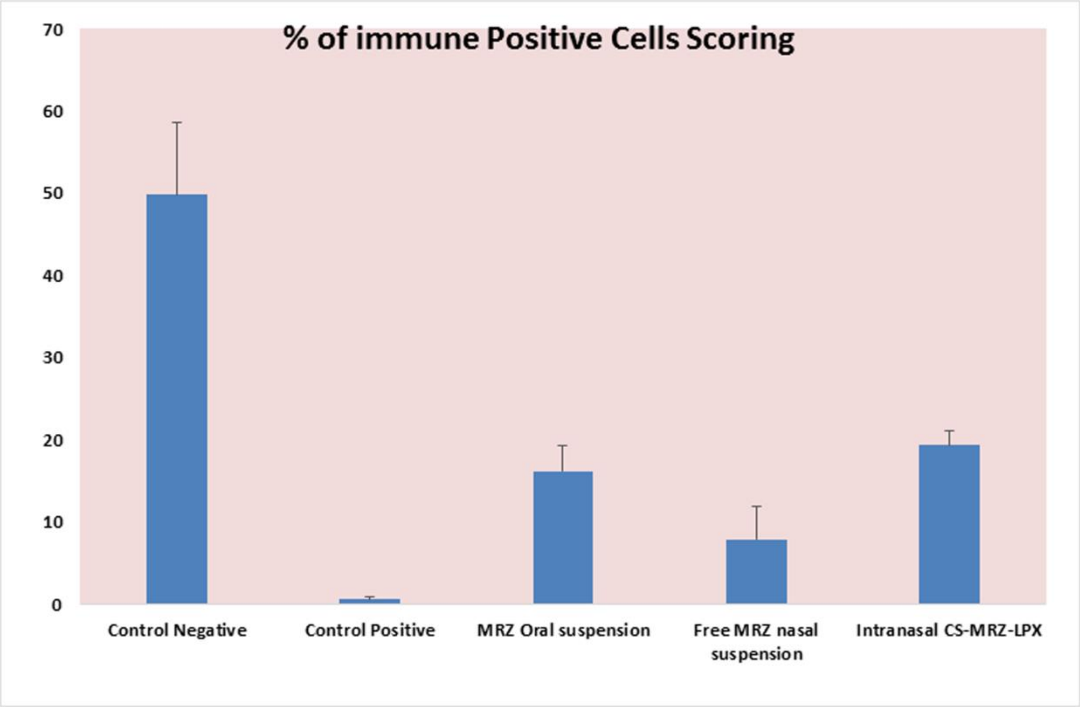

Supplementary Figure S5: Scoring of the immunohisto chemical analysis for the studied group in the pharmacodynamics study
